# Study on extracellular matrix metalloproteinase inducer and human epidermal growth factor receptor-2 protein expression in papillary thyroid carcinoma using a quantum dot-based immunofluorescence technique

**DOI:** 10.3892/etm.2015.2287

**Published:** 2015-02-12

**Authors:** TIAN TANG, DUAN-LIAN ZHANG

**Affiliations:** Department of Oncology, Renmin Hospital of Wuhan University, Wuhan, Hubei 430060, P.R. China

**Keywords:** papillary thyroid carcinoma, lymph node metastasis, extracellular matrix metalloproteinase inducer/human epidermal growth factor receptor-2, immunofluorescence

## Abstract

The aim of the present study was to investigate the association of extracellular matrix metalloproteinase inducer (EMMPRIN) and human epidermal growth factor receptor (HER)-2 protein expression in papillary thyroid carcinoma with lymph node metastasis (LNM), as well as the correlation between the two types of protein expression. A quantum dot-based immunofluorescence technique was used to detect EMMPRIN and HER-2 protein expression in 75 papillary thyroid carcinoma cases (including 70 cases of papillary thyroid carcinoma tissues and 5 cases of peri-tumor tissues). The positive rate and expression of EMMPRIN and HER-2 were compared and observed. The positive rate of EMMPRIN was 75.71% in papillary thyroid carcinoma tissues and 20.00% in peri-tumor tissues (P<0.05). The positive rate of HER-2 was 45.71% in papillary thyroid carcinoma tissues and 0% in peri-tumor tissues (P>0.05). The expression of EMMPRIN and HER-2 in papillary thyroid carcinoma was significantly associated with LNM (P<0.05). In addition, in the 70 papillary thyroid carcinoma tissues, the expression of EMMPRIN and HER-2 was positively and significantly correlated. In conclusion, this study demonstrates that the co-evolution of EMMPRIN and HER-2 may promote the occurrence and development of papillary thyroid carcinoma and LNM.

## Introduction

Thyroid cancer is one of the most common tumors derived from endocrine cells and its incidence rate has been rising (1). In order to improve the early diagnosis and treatment of patients, an in-depth study of the pathogenesis of thyroid cancer is urgently required.

Extracellular matrix metalloproteinase inducer (EMMPRIN) belongs to the immunoglobulin super family, which can promote tumor invasion and metastasis (2). Human epidermal growth factor receptor (HER)-2 gene is a type of proto-oncogene. As *in vivo* studies have shown, HER-2 oncogene is a neoplasm metastasis driving factor; malignant tumors with overexpressed HER-2 have a higher risk for tumor invasion and metastasis, with a poorer prognosis (3). Little has been reported on the detection of EMMPRIN and HER-2 protein expression in papillary thyroid carcinoma by the quantum dot (QD)-based immunofluorescence technique in tissue chips. In the current study, a QD-based immunofluorescence tissue analysis technique was adopted to detect EMMPRIN and HER-2 protein expression in tissue chips of papillary thyroid carcinoma from human sources. The correlation between the expression of EMMPRIN and HER-2 proteins was investigated in order to further understand the mechanism of the occurrence, development and prognosis of papillary thyroid carcinoma.

## Materials and methods

### Chips

Four tissue chips of papillary thyroid carcinoma were provided by Fanpu Biotech, Inc. (Guilin, China). These had a dot matrix of 15×10, a dot diameter of 1.1 mm and thickness of 4 μm, and included 70 cases of papillary thyroid carcinoma tissues and five peri-tumor tissues, in a dual chip matrix. Tissues were surgically resected during clinical surgery and then fixed for 4 h with neutral buffered formaldehyde. Among the patients with papillary thyroid carcinoma, there were 22 male and 48 female cases (age range, 25–80 years; mean, 57.2 years). There were 42 cases with lymph node metastasis (LNM) and 28 cases of papillary thyroid carcinoma tissues without LNM. The diagnosis of the patients was according to the World Health Organization Classification of papillary thyroid carcinoma (4).

### Antibodies

The primary antibodies were rabbit anti-human EMMPRIN polyclonal antibody (#sc-13976) and mouse anti-human HER-2 polyclonal antibody (#sc-33684; Santa Cruz Biotechnology Inc., Dallas, TX, USA; concentration, 1:100). The secondary antibodies were goat anti-rabbit/mouse biotinylated secondary antibodies (#AS-28175-05; AnaSpec, Inc., Fremont, CA, USA).

### Immunofluorescence

A QD-based immunofluorescence tissue chemical technique was used to detect EMMPRIN and HER-2 protein expression in tissue chips of papillary thyroid carcinoma. A hypersensitive fluorescence quantum dot kit containing Qdot Streptavidin Conjugate (QDs-SA) was purchased from Wuhan Jiayuan Quantum Dot Technological Development Co., Ltd. (Wuhan, China).

### Experimental procedures

The experimental procedures were carried out in strict accordance with the manufacturer’s instructions. Tissue chips of papillary thyroid carcinoma (thickness, 4 μm) were dewaxed, hydrated, microwave-antigen retrieved and washed with Tris-buffered saline (TBS). The tissue chips were blocked by incubation with blocking buffer solution (Beyotime Institute of Biotechnology, Shanghai, China) in a wet chamber for 30 min at 37°C. The tissue chips were then incubated with primary antibodies for 2 h at 37°C, then washed with TBS-Tween^®^ three times, 5 min/time. Supplement of secondary antibodies incubated for 1h at 37°C, washed repeatedly with TBS-Tween^®^ three times, 5 min/time. The tissue chips were again blocked by incubation with blocking buffer solution in a wet chamber for 30 min at 37°C. QDs-SA diluted with blocking buffer solution was dripped onto the tissue chips and incubated in a wet chamber for 30 min at 37°C. They were then washed three times with TBS-Tween for 5 min/time, and finally treated with 90% glycerin buffer. Following the addition of 900 ml/l glycerin, the chips were observed with an Olympus IX71 fluorescence microscope (Olympus Corporation, Tokyo, Japan). QDs were excited by irradiation at an excitation wavelength of 430–500 nm and an emission wavelength of 605 nm. When observed under the microscope, cells with reddish-orange fluorescent particles were positive. When the positive area was ≥25%, it was regarded as positive protein expression. In the control group, the primary antibody was substituted with TBS. When observed under the microscope, the positive staining of EMMPRIN and HER-2 protein was mainly located in the cell membrane and cytoplasm. Four different views were obtained under high power fields, and 200 cells were evaluated. Calculation of the degree of expression was conducted according to the percentage of positive cells: ‘−’, negative; ‘+’, ≤10% positive cells; ‘++’, 11–50% positive cells; and ‘+++’, ≥51% positive cells. Positive expression was deemed to be ≥11% positive cells.

### Statistical analysis

The quantitative analysis of QD staining was expressed as the mean ± standard deviation. One-way analysis of variance and Student-Newman-Keuls (q) detection were carried out with SPSS software, version 13.0 (SPSS Inc., Chicago, IL, USA) for positive cells in each staining group (α=0.05). Spearman’s rank correlation coefficient was adopted to determine the association between EMMPRIN and HER-2 expression. P<0.05 was considered to indicate a statistically significant difference.

## Results

### EMMPRIN protein expression

**Figure 1:**
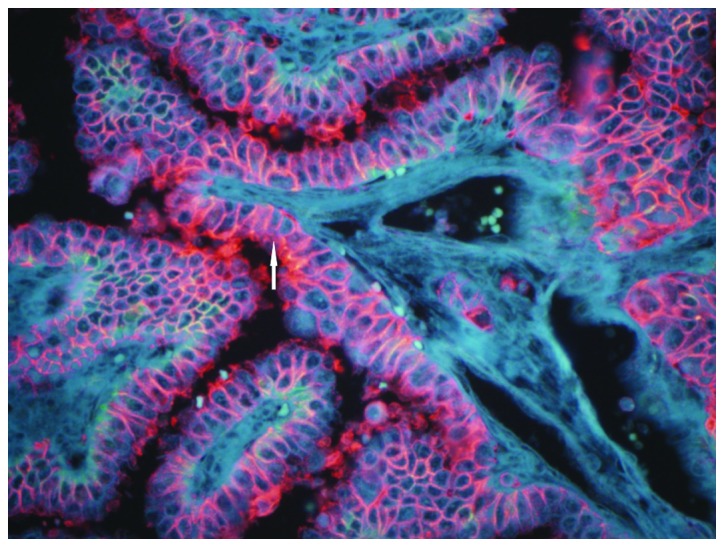
Expression of EMMPRIN protein in papillary thyroid cancer. A strong red fluorescence was seen in the cell membrane or cytoplasm of papillary thyroid cancer; the expression of EMMPRIN protein was strongly positive (white arrow). Quantum dot staining (magnification, ×200). EMMPRIN, extracellular matrix metalloproteinase inducer.

**Figure 2:**
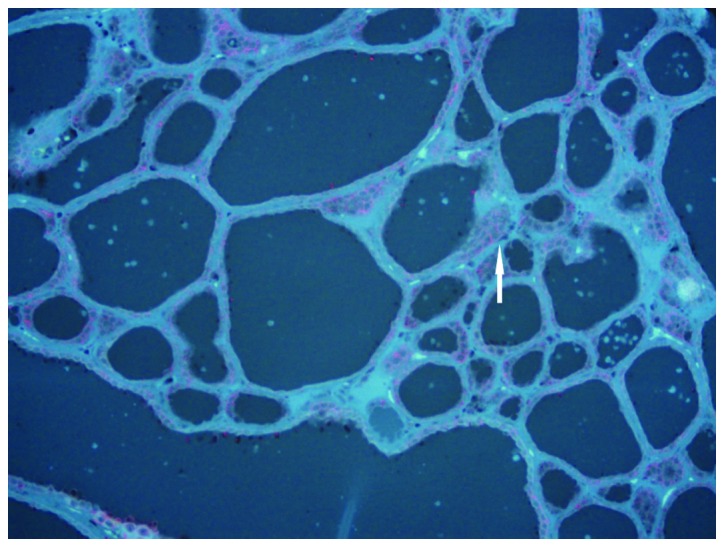
Expression of EMMPRIN protein in peri-tumor tissue. A small amount of red fluorescence was seen in the cell membrane or cytoplasm of the tissue adjacent to the cancer (white arrow); the expression of EMMPRIN protein was negative. Quantum dot staining (magnification, ×200). EMMPRIN, extracellular matrix metalloproteinase inducer.

Strong and red fluorescence of EMMPRIN was observed in the cell membrane or cytoplasm of papillary thyroid carcinoma (Fig. 1). A slight red fluorescence appeared in peri-tumor tissues; EMMPRIN protein exhibited negative expression (Fig. 2). The positive rate of EMMPRIN protein was 75.71% in papillary thyroid carcinoma and 20.00% in peri-tumor tissues (P<0.05; Table I). The positive rate of EMMPRIN protein in carcinoma tissues with LNM was significantly higher than that in carcinoma tissues without LNM (80.95 vs. 60.71%, P<0.05; Table II).

### HER-2 protein expression

**Figure 3:**
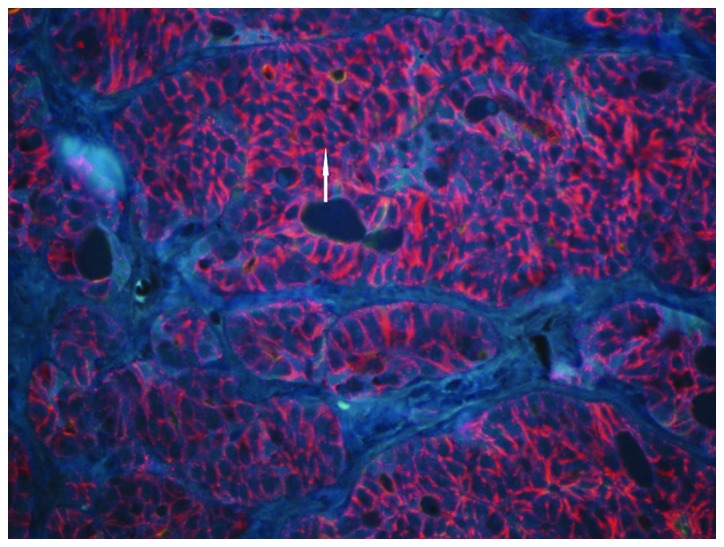
Expression of HER-2 protein in papillary thyroid cancer. A strong red fluorescence was seen in the cell membrane or cytoplasm of papillary thyroid cancer (white arrow); the expression of HER-2 protein was strongly positive. Quantum dot staining (magnification, ×200). HER, human epidermal growth factor receptor.

**Figure 4:**
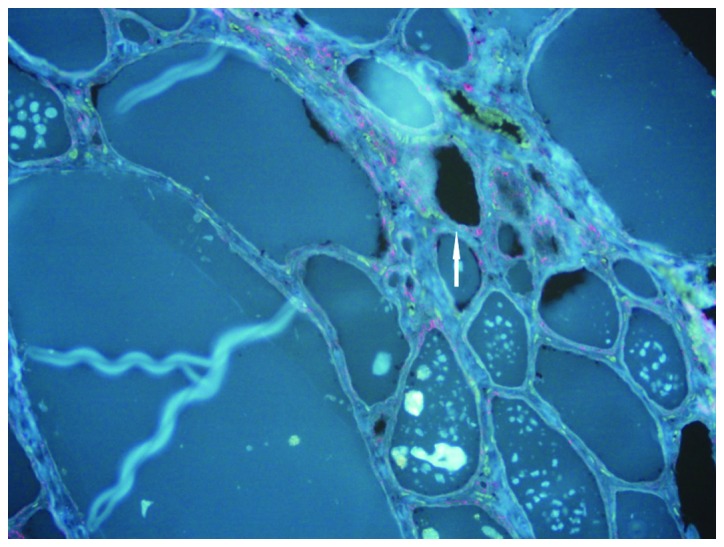
Expression of HER-2 protein in peri-tumor tissue. A small amount of red fluorescence was seen in the cell membrane or cytoplasm of the tissue adjacent to the cancer (white arrow); the expression of HER-2 protein was negative. Quantum dot staining (magnification, ×200). HER, human epidermal growth factor receptor.

Red fluorescence was observed in the cell membrane or cytoplasm of papillary thyroid carcinoma, which indicated positive HER-2 protein expression (Fig. 3). A slight red fluorescence appeared in peri-tumor tissues; HER-2 protein expression was negative (Fig. 4). The positive rate of HER-2 protein expression was 45.71% in papillary thyroid carcinoma and 0% in peri-tumor tissues (P<0.05; Table I). The positive rate of HER-2 protein expression in carcinoma tissues with LNM was significantly higher than that in carcinoma tissues without LNM (59.52 vs. 28.57%, P<0.05; Table II).

### Correlation between EMMPRIN and HER-2 protein expression in papillary thyroid carcinoma

Among 70 cases of papillary thyroid carcinoma tissues, 53 cases exhibited a positive expression of EMMPRIN protein and 32 cases were positive for HER-2 protein. There were 17 cases negative for EMMPRIN protein and 38 cases negative for HER-2. EMMPRIN and HER-2 had a positive correlation (r=0.375, P=0.001).

## Discussion

Biochip technology, as a biological research technique that emerged in the 1980s, generally includes gene and protein chips. Gene and protein chips are widely applied in molecular/synthetic biology to improve the development of a growing number of new functional genes and proteins. In order to further explain their function, it is necessary to turn to histology and histomorphological studies, which play a pivotal role in the study of cancer and require urgent development (5).

Currently, tissue slices are widely applied in medical studies, generally containing one (type of) tissue sample in one wax mass, that is, a single tissue slice. In scientific studies on large samples, a large number of tissue wax masses and tissue slices are prepared by repetitively carrying out the same procedure, resulting in a waste of time and resources, unavoidable errors and poor comparability. It is, therefore, necessary to design new multi-tissue embedding chip-making techniques, simplify the experiment times, reduce the workload of experiments and improve the efficiency. Tissue chips have the characteristics of small volume and high information content, as well as providing substantial results in a one-off experiment. In the present study, a 150-chip papillary thyroid carcinoma tissue chip in a dual-chip matrix was adopted. The experiment was completed with only a few chips, saving funds and reducing work load. Data from EMMPRIN and HER-2 protein expression in papillary thyroid carcinoma were obtained in a short time. Compared with traditional pathological techniques and methods, the results available are uniform, reliable and comparable, saving time and money in addition to providing a large quantity of information. This presents great potential for the future application of tissue chips in medicine (6,7).

QDs, made up of Group II–IV or Group III–V elements, are a type of semiconductor nanoparticle (diameter, 2–6 nm) capable of producing fluorescence upon laser excitation. Due to their special structure and unique optical properties, such as a broad and continuously distributed excitation spectrum, and narrow and symmetrically distributed emission spectrum, QDs have been widely used in medical studies. The size of QD kernels can be changed to precisely regulate the length of optical waves; thus, QDs have become a powerful tool in biomedical tagging and optical imaging (8). QDs, as new nanometer fluorescence probes, are characterized by high efficiency, wide coverage of light and stable optical properties. They are uniquely advantageous in live action monitoring and as long-term *in vivo* tracers (9). In recent years, nanometer-sized fluorescence probes with biological compatibility, developed on the basis of semiconductor QDs, have been provided with a unique optical property. QDs are promising in terms of application in the field of biomedicine, particularly in studies of biomedical imaging technology.

Tumors have a unique disease progession, involving invasion and metastasis. This is a complex process, with the following steps: Firstly, tumor cells break away from the primary tumor lesion, intrude into the mesenchyme and break down the base membrane to infiltrate and adhere to the outside of the vessel and intravasate into the vascular endothelium; secondly they enter the blood circulation system through the vascular endothelium, evading the immune response; thirdly, the tumor cells reach a new location through circulation and pass through the vascular wall and base membrane and complete extravasation into the extracellular matrix. Ultimately they survive, clone and proliferate in particular organs to form metastases.

EMMPRIN is a type of multifunctional protein, capable of inducing the production of matrix metalloproteinase (MMP) and promoting tumor cell invasion and metastasis (10).

The role of EMMPRIN, also known as CD147, in the process of tumor development, invasion and metastasis is a popular topic in the field of the cellular biology of tumors (11). The proliferative activity of tumor cells is closely associated with tumor invasion (12), metastasis (13) and prognosis (14).

In the present study, the expression rate of EMMPRIN in peri-tumor tissues was 20.00%, while it was 75.71% in papillary thyroid carcinoma tissues (P<0.05). EMMPRIN is highly expressed in papillary thyroid carcinoma tissues. The enhanced expression of EMMPRIN in papillary thyroid carcinoma tissues indicates that it could play a role in the occurrence and development of papillary thyroid carcinoma (11,15).

HER-2, also referred to as c-erbB-2, is a type of oncogene with an homologous sequence with the virus oncogene as well as the oncogene of epidermal growth factor receptor (EGFR), and is overexpressed in various types of tumor (16,17). HER-2 can form compounds (such as heterodimers) with other members of the EGFR family, such as EGFR erbB1/HER1, erbB3/HER3 and erbB4/HER4, engage in cell proliferation signal transduction, and finally result in cell proliferation or even carcinomatosis (18). HER-2 can be slightly expressed in coelomic epithelium and glandular epithelium tissues, although without gene amplification, and its overexpression is associated with cellular carcinomatosis. Therefore, the detection of overexpressed HER-2 protein can indicate HER-2 oncogene amplification indirectly (19–22). HER-2 overexpression plays an important role in the occurrence and development of certain forms of cancer and has effects on tumor progression and therapy (23).

In the current study, the positive rate of EMMPRIN protein expression in tumor tissues with LNM was found to be 80.95% (34/42), significantly higher compared with the EMMPRIN protein expression in tumor tissues without LNM at 60.71% (17/28) (P<0.05). This shows that EMMPRIN protein expression is closely associated with LNM. The result corresponds well with the previously reported literature (24,25).

EMMPRIN shows high expression in papillary thyroid carcinoma; however, only a few factors inducing its expression in the development of tumors are known (26). Amphiregulin and EGF serve as regulation factors, which are able to induce the expression of EMMPRIN by the activation of protein tyrosine kinase of EGFR (27). In addition, anti-EGFR antibody can inhibit EMMPRIN expression and MMP activity (28). This demonstrates that EGFR signaling may play a decisive role in the regulation process. In the present study, it was found that EMMPRIN and HER-2 were concurrently expressed in papillary thyroid carcinoma tissues; the rate of co-expression was 45.71% (32/70). EMMPRIN is positively and significantly associated with HER-2. EMMPRIN and HER-2 could jointly control MMP activity and collaboratively promote the LNM of papillary thyroid carcinoma, therefore being jointly involved in the occurrence, development and metastasis of papillary thyroid carcinoma. This result shows that the development of a tumor is a multi-factorial, multi-stage process.

EMMPRIN and HER-2 exhibit positive expression in papillary thyroid carcinoma, which is substantially associated with LNM. Further study on EMMPRIN and HER-2 inhibitors may serve as a new approach for inhibiting the invasion and metastasis of papillary thyroid carcinoma and, most significantly, may improve the prognosis of patients with papillary thyroid carcinoma.

## Figures and Tables

**Table I:** Expression of EMMPRIN and HER-2 in thyroid papillary carcinoma and adjacent (peri-tumor) tissues.

		EMMPRIN		HER-2	
			
Group	n	−	+	−	+
Thyroid papillary carcinoma	70	17	53	38	32
Peri-tumor tissue	5	4	1	5	0

EMMPRIN, extracellular matrix metalloproteinase inducer; HER, human epidermal growth factor receptor.

**Table II:** Expression of EMMPRIN and HER-2 protein and the association with LNM.

		EMMPRIN		HER-2	
			
LNM	n	−	+	−	+
Yes	42	8	34	17	25
No	28	11	17	20	8

EMMPRIN, extracellular matrix metalloproteinase inducer; HER, human epidermal growth factor receptor; LNM, lymph node metastasis.
